# Supplementary material for: Identification of Freezing-Responsive microRNAs and Their Targets in Chinese Jujube by Small RNA and Degradome Sequencing
Source: Int J Mol Sci. 2026 Sep 4;27(17):7912. doi: 10.3390/ijms27177912 (PMC13566890; doi:10.3390/ijms27177912)
Supplement: Supplementary file 1 [file ijms-27-07912-s001.zip › Supplementary Material S2/novel_miR_155.pdf]

Provisional ID : novel_miR_155
Score total : 0.5
Score for star read(s) : -1.3
Score for read counts : 0
Score for mfe : 0.2
Score for randfold : 1.6
Score for cons. seed :
Total read count : 88
Mature read count : 87
Loop read count : 1
Star read count : 0

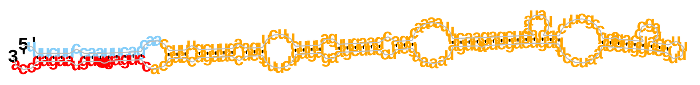

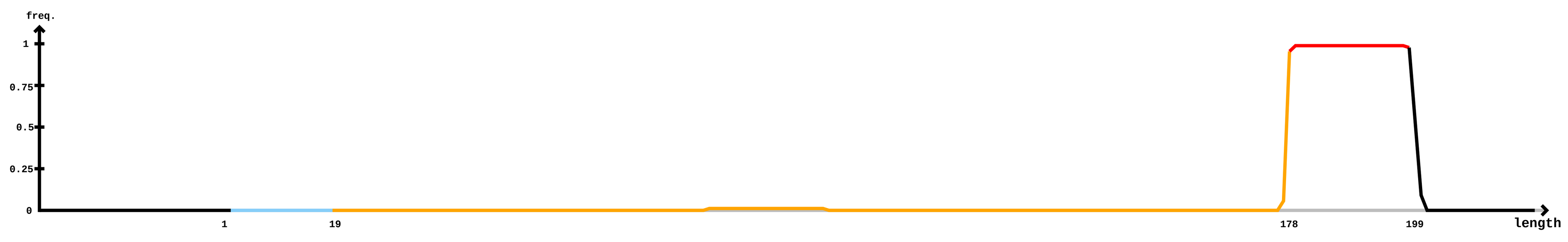

Star Mature

```
5'- cacugacuuccuccugcagcuuugacuugauuucuuccaauucagcaaccuuugcuugaaguucuucuuuaguugaagcagccaaaauuucaagagcugaaauauccuccuucgccugagacuccgaaagcuuuugcugguccaaauccugagucagcucauugauaaaagcuuccuucgaagagagucuuugcuccaagucaagcaccugagauuuuguaagagccagcuccucuuggacugcagaaag -3'  exp
    ............((((((((..((.(((..(((((.((((((((....(((.(((((.(((....((((..((((((.(((.......(((((((((((......(((.......((.((((.....(((....)))))))))......)))))))))).)))).....)))..))))))..)))).....))).))))).)))...)))))...))).))))).))).)).....)).))))))....  reads  mm  sample
    .....................................................................................................................uucgccugagacuccgaaag..............................................................................................................  1  0  LT-1
    .................................................................................................................................................................................accugagauuuuguCagagccagc....................  1  1  LCK-3
    .................................................................................................................................................................................accugagauuuuguaagagccagc....................  4  0  LCK-3
    ..................................................................................................................................................................................ccugagauuuuguaagagcc......................  1  0  LT-3
    ..................................................................................................................................................................................ccugagauuuuguaagagcca.....................  46  0  LCK-1
    ..................................................................................................................................................................................ccugagauuuuguaagagcca.....................  32  0  LCK-2
    ...................................................................................................................................................................................cugagauuuuguaagagccagc...................  3  0  LT-3
```
